# Pharmaceutical supply chain risk assessment in Iran using analytic hierarchy process (AHP) and simple additive weighting (SAW) methods

**DOI:** 10.1186/s40545-015-0029-3

**Published:** 2015-02-28

**Authors:** Mona Jaberidoost, Laya Olfat, Alireza Hosseini, Abbas Kebriaeezadeh, Mohammad Abdollahi, Mahdi Alaeddini, Rassoul Dinarvand

**Affiliations:** Department of Pharmacoeconomics and Pharmaceutical Administration, Faculty of Pharmacy, Tehran University of Medical Sciences, Tehran, 1417614411 Iran; Management Department, Allame Tabatabaei University, Tehran, Iran; Food and Drug Organization, Ministry of Health and Medical Education, Tehran, Iran; Department of Toxicology and Pharmacology, Faculty of Pharmacy and Pharmaceutical Sciences Research Center, Tehran University of Medical Sciences, Tehran, Iran; Department of Industrial Engineering, University of Science and Culture, Tehran, Iran; Department of Pharmaceutics, Faculty of Pharmacy, Tehran University of Medical Sciences, Tehran, Iran

**Keywords:** Pharmaceutical supply chain, Risk assessment, analytic hierarchy process (AHP) method, Simple additive weighting (SAW) method, Iran pharmaceutical industry

## Abstract

**Objectives:**

Pharmaceutical supply chain is a significant component of the health system in supplying medicines, particularly in countries where main drugs are provided by local pharmaceutical companies. No previous studies exist assessing risks and disruptions in pharmaceutical companies while assessing the pharmaceutical supply chain. Any risks affecting the pharmaceutical companies could disrupt supply medicines and health system efficiency. The goal of this study was the risk assessment in pharmaceutical industry in Iran considering process's priority, hazard and probability of risks.

**Methods:**

The study was carried out in 4 phases; risk identification through literature review, risk identification in Iranian pharmaceutical companies through interview with experts, risk analysis through a questionnaire and consultation with experts using group analytic hierarchy process (AHP) method and rating scale (RS) and risk evaluation of simple additive weighting (SAW) method.

**Results:**

In total, 86 main risks were identified in the pharmaceutical supply chain with perspective of pharmaceutical companies classified in 11 classes. The majority of risks described in this study were related to the financial and economic category. Also financial management was found to be the most important factor for consideration.

**Conclusion:**

Although pharmaceutical industry and supply chain were affected by current political conditions in Iran during the study time, but half of total risks in the pharmaceutical supply chain were found to be internal risks which could be fixed by companies, internally. Likewise, political status and related risks forced companies to focus more on financial and supply management resulting in less attention to quality management.

## Introduction

Medicines Supply is one of the major priorities in developing countries. Therefore efficient pharmaceutical supply chain management is of high importance [1,2]. The efficient pharmaceutical supply chain supplies medicines in the right quantity, and to the customers with the acceptable quality, at the proper time and with optimum price to produce benefits for all the stakeholders [2,3]. The pharmaceutical supply chain is a significant component of the health scheme which includes all procedures, information, resources and players such as suppliers, manufacturers, intermediaries, third-party service providers, logistics activities, merchandising and sales activities, finance and information technology [2,4]. Pharmaceutical companies play an important role in supplying medicines, particularly in countries where the volume is supplied by local companies [5]. Pharmaceutical companies in Iran are important players in the supply chain as more than 95% of drug market in Iran (in terms of volume) is supplied by local manufacturers [6]. In this context, any risks affecting the pharmaceutical companies could disrupt the supply of medicines and affect the health system efficiency [7,8].

Local pharmaceutical companies face several risks and vulnerabilities due to nature of Pharmaceutical industry and its complex processes in one hand and political condition of Iran in the other hand [2,6,9,10]. In addition, medicine is a highly regulated good and under the controls and restrictions of public regulatory authorities [2,11,12]. To overcome supply chain vulnerabilities, it is essential to identify and prioritize obstacles to create best practice for proper configuration and adaptability in pharmaceutical industry [13,14].

Supply chain risk management can help organizations to monitor the expecting hazards and control possible risks and thus improve efficiency of the supply chain [15]. This approach can help to improve and build up business processes, prevent potential problems, minimize loss of commercial enterprise, reduce costs and liability, protect the supply chain and avoid waste [16,17].

Referring to International Conference on Harmonization (ICH) Q9 definition; risk assessment is defined as “A systematic process of organizing information to support a risky decision to be made within a risk management process. It consists of the identification of hazards and the analysis and evaluation of risks associated with exposure to those hazards.”

The risk identification stage represents an important initiating point in the overall risk management process and forms the basis for the next stages [18]. Potential hazards which are outputs from the risk identification stage will be subject to detailed scrutiny during the hazard analysis and evaluation stages [19,20]. At the following step as risk analysis, level of risk in terms of severity of hazard, the likelihood of occurrence and detection should be estimated that provides a quantitative idea of each risk. And at the last step of risk assessment, output of risk analysis should be organized (filtered, ranked and etc.) and most significant risks should be highlighted and identified for risk management strategies [21-23].

There are several reports which report risks in the pharmaceutical supply chain and some of them attempted to quantify or measure them [2,6,16,24]. Supply and supplier issue, fragmentation, delivery reliability, information flow, quality management system, inventory management, customer service disruption, research and development, skill of workers, planning, organization and processes, company strategies, production cost and waste management, fiscal management, currency rate, logistic, demand, regulations are some topics which were described in previous studies as source of risks for pharmaceutical companies. The objectives of this study were to identify and categorize pharmaceutical supply chain risks with perspective of local companies. In addition, main risks of supply chain functions in local companies were measured considering priority of functions, hazard and probability of risks in pharmaceutical supply chain.

## Methods

This study was carried out in four phases; risk identification through literature review (carried out on September 2012), risk identification in Iranian pharmaceutical companies through interview with experts, risk analysis through questionnaire, risk evaluation (carried out from November 2012 till December 2013).

*Phase 1:* A literature review to identify pharmaceutical supply chain risks; a systematic review via scientific search engines, Scopus, PubMed, Web of Science and Google scholar, was done for risk identification by several keywords; Supply chain management, risk, risk management, risk assessment, pharmaceutical, pharmaceutical industry, Iran. Also investigating in each data base was done based on data base characteristics and Medical Subject Headings (MeSH) was noted when searching via PubMed.

In addition, categorization of pharmaceutical supply chain processes and functions was investigated in the literatures, in this phase.

All studies were screened through 4 steps:

- Reviewing reports and non-relevant articles were excluded considering outcome of interests and scope of the study;
- Excluding duplicated articles;
- Excluding non-relevant articles, based on abstract review
- Finally, full texts of all remained studies were investigated and some of articles were excluded by outcome of interest.

This study focused on pharmaceutical supply chain risk management with perspective of the production manufacturers so, studies with consumer safety, environmental risk management, health policy and third parties’ subject were eliminated.

*Phase 2:* Risk identification in Iran pharmaceutical companies through expert opinion with an open questionnaire; for identifying pharmaceutical supply chain risks in Iran, investigation risks through expert opinion was carried out. In this step, a group of 16 experts who had at least 5 years experience in pharmaceutical industry management was selected to interview for risk identification. It is trying to select experts from different kind of companies and organizations with different ownerships and field of work to cover all points of view in the Iran’s pharmaceutical industry.

Before each interview, an introductory letter and open questionnaire, which was validated in the pilot study, were sent to experts. 1–2.5 hour interview with each expert was carried out and the questions related to risks and best practice for categorization of pharmaceutical supply chain processes and functions were asked. After coding and extracting risks from questionnaires, a summary of interview and identified risks were sent to interviewees for review and final confirmation.

*Phase 3:* Risk analysis through a questionnaire and interview with experts; all risks extracted from literature review and expert interview (phase 1 and 2) collected in a questionnaire for risk analysis. The questionnaire was designed in two parts with two tables; first part included a table for pair wise comparison to prioritize supply chain functions based on group AHP method and the second part included a table for scoring hazard of risks on supply chain functions and probability of risks in Iran pharmaceutical industry simultaneously by 0–10 rating scale.

A questionnaire was validated by 3 experts in the pilot study before sending a questionnaire to the experts. After sending questionnaires to the experts, an interview meeting was set with each expert to fill questionnaire in the face to face interview meeting. Except one, all questionnaires were responded by the expert team.

**Figure 1:**
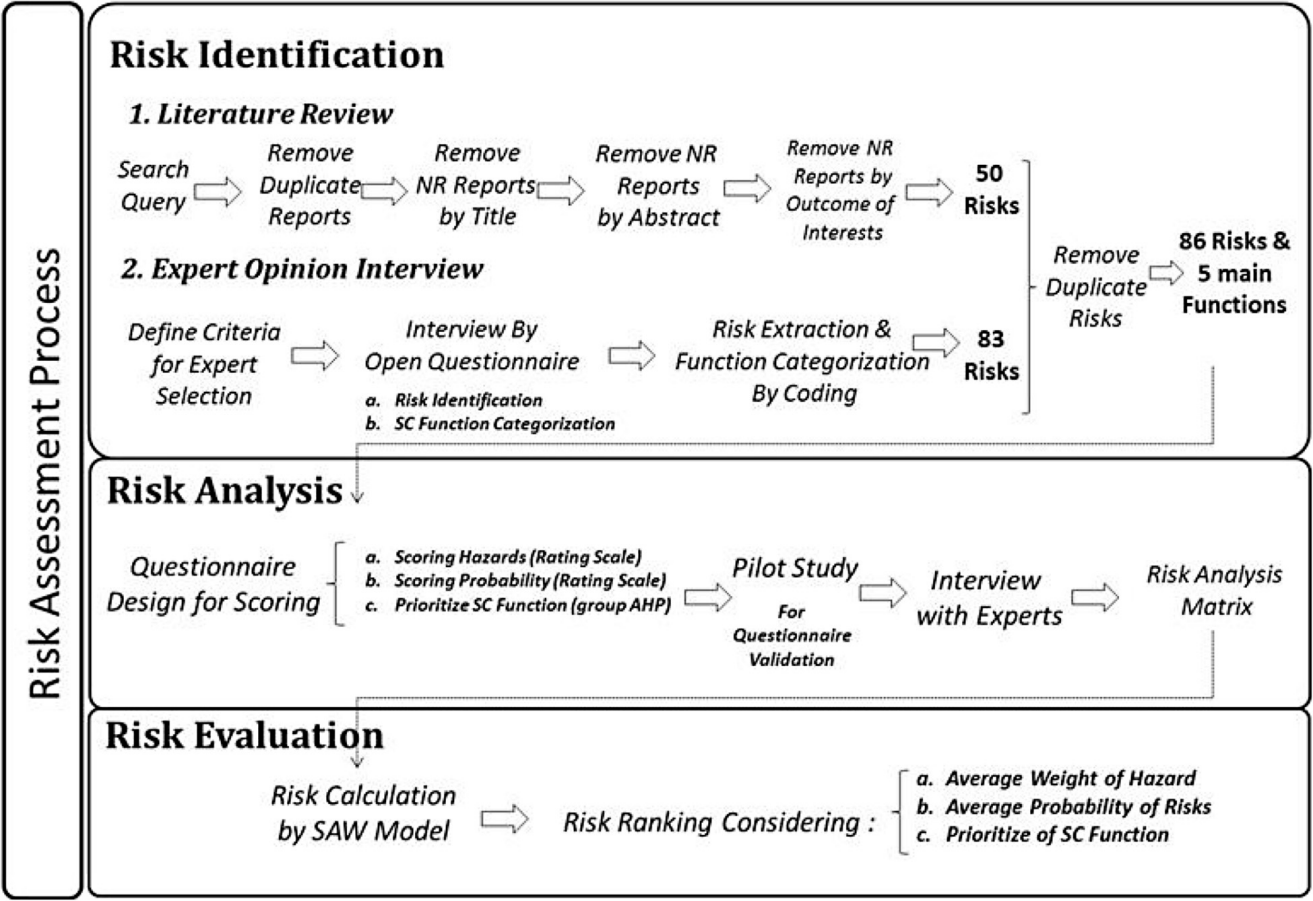
**Risk assessment process.**

*Phase 4:* Risk evaluation; risk evaluation was based on considering three factors; probability of risks, hazards of risks on each function in supply chain and priority of managing supply chain functions. AHP group decision making method was selected for prioritizing functions in the supply chain and rating scale was selected for scoring hazard of risks on functions of the supply chain and probability of risks in Iran pharmaceutical industry. After gathering the data, simple additive weighting (SAW) method was applied for evaluation. Figure 1 shows a risk assessment process which is applied in this study.

*Rating scale:* A rating scale is widely used in studies by applying questionnaires to elicit information about a quantitative or a qualitative attribute. In this method, a person selects the number which is considered to reflect the intensity to an item [25].

*AHP group decision making:* AHP is a type of multi attribute decision making (MADM) introduced by Thomas L Saaty (1971) which helps in complex decision making involving multiple scenarios, criteria and actors [26]. AHP is selected because it allows decision-makers to model a complex problem in a hierarchical structure with considering goals and criteria [27]. In AHP, pairwise comparisons are used to determine preferences between criteria or alternatives [28] (Table 1).

**Table 1:** **The AHP Pair-wise comparison values or scale of preference between two elements**

**Preference weights or level of importance (value of a**_**ij**_ **)**	**Definition of verbal scale**	**Explanation**
a_ij_ = 1	If the two criteria are equally preferred	Two activities or criteria contribute equally to the objective or goal
a_ij_ = 3	If criteria i is moderately preferred than criteria j	Experience and judgment slightly favor criteria over another
a_ij_ = 5	If criteria i is strongly preferred than criteria j	Experience and judgment strongly favor one criteria over another
a_ij_ = 7	If criteria i is very strongly preferred than criteria j	An criteria is strongly favored over another and its importance demonstrated in practice
a_ij_ = 9	If criteria i is absolutely preferred than criteria j	The evidence favoring one criteria over another is of the highest degree possible of affirmation
2,4,6,8	Intermediate values	Use to compromise between two judgments

In this study group, decision AHP is applied in which comparative judgment of all decision makers gathered and aggregated. So, using aggregation functions in group AHP method makes it more applicable than AHP [29]. Four basic steps are needed to apply the AHP method; hierarchy construction, individual comparative judgments (pairwise comparison), aggregation of judgment by the geometric average between each judgment of decision makers, and then synthesizing the results [30].

Typically, hierarchy is structured from the top (goal) to the end (criteria or alternative) which the goal is finding the most appropriate criteria or alternative among others based on the weight of each criteria or alternative. In fact the group AHP method based on the pairwise comparison tries to find differences between criteria or alternatives and obtain the weight of each objective. The goal of this research was to evaluate and prioritize management of supply chain functions from a pharmaceutical production company perspective. Below steps are defined based on the group AHP approach, to prioritize pharmaceutical supply chain functions:

1. Hierarchy construction: hierarchy structure of supply chain functions which categorized into 5 group ups by experts was drawn (Figure 2).

**Figure 2:**
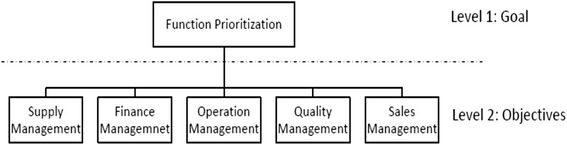
**Hierarchy structure of pharmaceutical supply chain risks.**
2. Comparative judgments (pairwise comparison): after developing the structure, pairwise comparisons were done by experts through questionnaires to determine the relative importance of the function management in the pharmaceutical supply chain.
3. Aggregation of comparative judgment: using geometric average
4. Synthesize the results to find the overall score of each function [31]

Simple additive weighting (SAW) method: SAW is a simple and most applicable multi-attribute decision method which is known as a weighted linear combination or scoring technique. This method is based on the weighted average and an evaluation score is measured by multiplying the normalized value of each criteria for the objectives with the importance of the criteria. Then the objectives could be ranked and objective with the highest score is selected as the preferred one [32].

Based on SAW technique, final score of each risk is calculated and ranked as follow [33]:

$$ {\mathrm{S}}_{\mathrm{i}}={\displaystyle \sum_{\mathrm{j}=1}^{\mathrm{M}}}{\mathrm{w}}_{\mathrm{j}}{\mathrm{r}}_{\mathrm{i}\mathrm{j}}\;\mathrm{f}\mathrm{o}\mathrm{r}\ \mathrm{i}=1,2,\dots,\ \mathrm{N} $$

Where

S_i_ is total score of the i^th^ objective;

r_ij_is the normalized rating of the i^th^ objective for the j^th^ criterion,

w_i_ is the importance (weight) of the i^th^ criterion;

N is the number of objectives;

M is the number of criterion;

Based on this method, final risk scores were calculated considering the average probability of risks, the average hazard of risks on each function and priority of supply chain functions.

Expert choice version 11 was applied for AHP analysis and Microsoft Excel was applied to calculations based on SAW method.

## Results

### Outcome of interest

Supply chain risk identification and assessment from pharmaceutical production company’ perspective was defined as the outcome of interest in this study. So, consumer safety, environmental, health policy and third party perspective were not considered.

*Phase 1:* based on the systematic review on risk identification which was done, after reviewing abstracts, none-relevant studies were excluded. Finally, 28 studies from Google scholar, 10 studies from Web of Science, 44 studies from Scopus and 12 studies from PubMed were evaluated. Full 94 studies were reviewed and 85 out of 94 were eliminated by outcomes of interest. At the end, 9 articles included in the analysis for risk extraction.

Out of these 9 studies, 50 “risks” were identified from the literature from the literature which categorized into 7 main categories; categories were systematic review; supply and supplier issues, organization and strategy issues, financial, logistical, political, market and regulatory issues. Most mentioned items in articles which reported as risk were related to supply and suppliers [33] (Table 2).

**Table 2:** **Risks identified through systematic review with their frequency of reporting**

**Category**	**Risks**	**No.**	**Category**	**Risks**	**No.**	**Category**	**Risks**	**No.**
Supply & suppliers issues	Supply and supplier issue	6	Supply & suppliers issues	Flexibility in product variety	1	Financial issues	Tax payable change	3
	Partnership with supplier	3		Timely delivery	1		Currency rate	3
	Raw material quality	2		Quality management system	1		Financial risks	3
	Ordering cycle time	2	Organization & strategies issues	Customer services disruption	1		Tariff policies changes	1
	Contract & agreements	2		Inventory management	4		Costs related to supply	1
	Customization of supplier	2		Operation issues	3		Cash Flow	1
	Certificate of good manufacturing practices (GMP)	2		R & D	2		Interest rate	1
	Flexibility of supplier	2		Skill of workers	2	Market issues	Market	2
	Fragmentation	1		Strategy	2		Consumers taste	2
	Delivery reliability	1		Planning issues	2		Demand	2
	Environmental assessment	1		Information flow	1	Political issues	Natural disasters & Terrorism	3
	Technology level	1		Visibility on stock	1		Political issues	1
	Information systems	1		Organization & process	2		Sanction	1
	Good will	1		Mergers and acquisition	1	logistic issues	Counterfeit	4
	Technology development	1		Time to market	1		Transportation	3
	Flexibility in delivering	1		waste management	1	Regulation	Regulation	6
	Flexible quantities	1		Production cost	1			

*Phase 2:* Totally 442 items as pharmaceutical supply chain risk were mentioned in interviews by experts. After coding and rewording items, 83 items were extracted and considered as “risks” in the pharmaceutical supply chain from production manufacturers perspective. Forty seven out of 83 mentioned risks were reported in the literatures in phase 1. And 36 items out of 83 were new items. After syncing risks from literature and interviews, a list of 86 risks which categorized in 11 groups were identified. Categorization of risks was based on expert opinions which include financial and economic, politics and government, regulatory, company strategies, research and development, supply and suppliers, market and competitors, operation and processes, logistic, human resource, and disaster and accidents.

Also, based on the expert’s opinion, supply chain functions in pharmaceuticals were categorized into five groups; financial management, supply management, operation management, sales and quality management.

*Phase 3*: Risk assessment was done by considering 3 factors in second questionnaire. These three factors were priority of supply chain functions, probability of risks and hazard of risks on each function.

**Figure 3:**
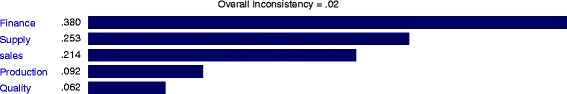
**Major decision objectives priorities.**

The pair wise comparison of the supply chain functions shown in Table 3 as well as in Figure 3 indicated that financial management is the most important function to be managed with a priority of 0.380 followed by supply management (0.253), sales management (0.214), operation management (0.092) and quality management (0.062). Overall inconsistency was in the acceptable range (0.02) (Figure 3).

**Table 3:** **All supply chain risks and ranks**

**Rank**	**Risk**	**Probability(%)**	**Weight**	**Rank**	**Risk**	**Probability(%)**	**Weight**
1	Sanctions	63.93	0.8543	11	Operational cost	53.57	0.5819
2	Money transfer	71.07	0.8036	12	Ordering cycle time	48.85	0.5732
3	Interest rate	60.36	0.7482	13	Material cost	45.36	0.5639
4	Currency fluctuation	62.50	0.7401	14	MOH policy fluctuation	57.86	0.5368
5	Cash flow	51.79	0.7158	15	Money collection	44.29	0.5154
6	Unstable policies	63.57	0.6766	16	Banking regulation	46.43	0.5142
7	Pricing policies	58.93	0.6616	17	Suppliers conditions	43.46	0.4841
8	Inflation rate	56.07	0.6411	18	Managerial knowledge	38.85	0.4812
9	Information flow	48.57	0.6190	19	Strategies	36.79	0.4703
10	Regulation transparency	56.43	0.6074	20	Economic stagnation	48.86	0.4670

Average of probability and hazard of risks of supply chain factors which scored by experts through rating scale, were measured. Then, based on SAW method, the weight of each risk considering the priority of supply chain functions (priorities were measured by AHP method), probability of risks and hazard of risks on each function (which obtained from expert opinion through rating scale) was calculated. Table 4 shows a list of risks with their rank, probability and weight.

**Table 4:** **Top 20 risks of financial management function**

**Rank**	**Risks**	**Weight**	**Rank**	**Risks**	**Weight**
1	Interest rate	1.0000	11	Operational cost	0.6728
2	Currency fluctuation	0.9019	12	Material cost	0.6000
3	Money transfer	0.8641	13	Regulation transparency	0.5881
4	Sanctions	0.8627	14	Ordering cycle time	0.5417
5	Cash flow	0.8372	15	Economic stagnation	0.5222
6	Inflation rate	0.7665	16	Information flow	0.4867
7	Pricing policies	0.7008	17	Suppliers conditions	0.4820
8	Unstable policies	0.6880	18	Return of investment	0.4810
9	Banking regulation	0.6762	19	Managerial knowledge	0.4568
10	Money collection	0.6746	20	Biased interpretation of regulations	0.4279

The results showed that the average probability of risks in the pharmaceutical supply chain in Iran is 35.05% and major risks are related to the financial and economical category with a weight of 0.5542, and followed politics and government (0.5171), regulatory (0.3911), strategies (0.3723), research and development (0.3247), supply and suppliers (0.2907), market and competitors (0.2868), operation and process (0.2765), logistic (0.2728), human resource (0.2462) and disaster and accidents (0.0896).51 out of 86 risks are external with an average probability of 36.59% and average weight of 0.3607 and probabilities of internal risk is 32.81% with average weight of 0.3397.

Considering probability and hazard of each risk and normalizing numbers, high important risks in each function of supply chain in pharmaceutical companies were measured as below:

Interest rate [1], currency fluctuation (0.9018), money transfer (0.8641), sanctions (0.8627), cash flow (0.8372), inflation rate (0.7664), pricing policies (0.7007), unstable policies (0.6880), banking regulation (0.6761) and money collection (0.6745) are top ten risks which have more impact on financial management function in Iran pharmaceutical supply chain. Table 5 shows top 20 risks with high impact on financial management function.

**Table 5:** **Top 20 risks of supply management function**

**Rank**	**Risks**	**Weight**	**Rank**	**Risks**	**Weight**
1	Money transfer	1.0000	11	Information flow	0.5289
2	Sanctions	0.8682	12	Inflation rate	0.5246
3	Currency fluctuation	0.7035	13	Commercial regulation	0.5170
4	Cash flow	0.6399	14	Limited suppliers	0.5125
5	Ordering cycle time	0.6275	15	MOH policy fluctuation	0.4743
6	Unstable policies	0.6145	16	Banking regulation	0.4601
7	Interest rate	0.6129	17	Biased interpretation of regulations	0.4317
8	Regulation transparency	0.5799	18	Customs regulations	0.4237
9	Material cost	0.5660	19	Supplier commitments	0.4047
10	Suppliers conditions	0.5371	20	Medicine regulations	0.4011

Money transfer (1), sanctions (0.8682), currency fluctuation (0.7035), cash flow (0.6399), ordering cycle time (0.6275), unstable policies (0.6144), interest rates (0.6129), regulatory transparency (0.5799), operational cost (0.5660) and suppliers’ conditions (0.5370) are top ten risks which have more impact on supply management function in Iran pharmaceutical supply chain. Table 6 shows top 20 risks with high effect on supply management function.

**Table 6:** **Top 20 risks of sales management function**

**Rank**	**Risks**	**Weight**	**Rank**	**Risks**	**Weight**
1	Pricing policies	1.0000	11	Operational cost	0.6636
2	Lobbing	0.8199	12	Currency fluctuation	0.6576
3	Inflation rate	0.7993	13	Cash flow	0.6503
4	Distribution & coverage	0.7883	14	New competitors (new medicine or new company)	0.6485
5	Unstable policies	0.7767	15	Interest rate	0.6350
6	Sanctions	0.7485	16	Time to market	0.6240
7	MOH policy fluctuation	0.7167	17	Managerial knowledge	0.6197
8	Information flow	0.6924	18	Strategies	0.6180
9	Regulation transparency	0.6895	19	Competition	0.6177
10	Product selection	0.6733	20	Demand forecasting	0.5939

Pricing policies (1), lobbying (0.8199), inflation rate (0.7992), distribution & coverage (0.7883), unstable policies (0.7767), sanctions (0.7485), (Ministry of Health) MOH policy fluctuation (0.7167), information flow (0.6924), regulatory transparency, (0.6895) and product selection (0.6733) are top ten risks which have more impact on sales management in Iran pharmaceutical supply chain. Table 7 shows top 20 risks with high effect on sales management function.

**Table 7:** **Top 20 risks of operation management function**

**Rank**	**Risks**	**Weight**	**Rank**	**Risks**	**Weight**
1	Sanctions	1.0000	11	Regulation transparency	0.6388
2	Information flow	0.9097	12	Motivation	0.6357
3	Money transfer	0.8192	13	Planning & ordering	0.6355
4	Operation standers	0.7574	14	Currency fluctuation	0.6303
5	Cash flow	0.7354	15	Medicine regulations	0.6252
6	Inappropriate production process and technology	0.6888	16	Location	0.6111
7	Unstable policies	0.6804	17	Raw material quality	0.5972
8	Ordering cycle time	0.6635	18	Human errors	0.5948
9	Operational cost	0.6616	19	Strategies	0.5906
10	MOH policy fluctuation	0.6430	20	Material cost	0.5881

Sanctions (1), information flow (0.9097), money transfer (0.8191), operation standers (0.7574), cash flow (0.7354), inappropriate production process and technology (0.6887), unstable policies (0.6803), ordering cycle time (0.6635), operational cost (0.6615) and MOH policy fluctuation (0.6430) are top ten risks which have an impact on operations management function in Iran pharmaceutical supply chain. Table 8 shows top 20 risks with high effect on operations management function.

**Table 8:** **Top 20 risks of quality management function**

**Rank**	**Risks**	**Weight**	**Rank**	**Risks**	**Weight**
1	Operation standers	1.0000	11	Human errors	0.7650
2	Raw material quality	0.9302	12	knowledge of regulatory people	0.7397
3	Information flow	0.9141	13	Motivation	0.7107
4	Inappropriate production process and technology	0.8248	14	Good storage practices (GSP)	0.7022
5	Sanctions	0.8081	15	Pricing policies	0.6952
6	Location	0.8070	16	Supplier quality specifications	0.6638
7	In Process quality	0.7897	17	Formulation	0.6606
8	MOH policy fluctuation	0.7801	18	Business awareness of regulators	0.6156
9	Skilled workers	0.7753	19	Strategies	0.5890
10	Medicine regulations	0.7720	20	Biased interpretation of regulations	0.5490

Operation standers (1), raw material quality (0.9302), information flow (0.9141) inappropriate production process and technology (0.8247), sanctions (0.8080), location (0.8070), in process quality (0.7897), MOH policy fluctuation (0.7800) and skilled workers (0.7752) are top ten risks which were found to have an impact on quality management function in Iran pharmaceutical supply chain. Table 9 shows top 20 risks with high effect on quality management function.

**Table 9:** **Risks probability-hazard chart**

	**High Probability**	**Low Probability**
**High hazard**	Sanction	Civil war
	Cash flow	In process contamination
	Information flow	Natural disasters
	Ordering cycle time	Accidents
		Counterfeit
**Low hazard**	Biased interpretation of regulations	Human resource turnover
	Government dependency	Supplier agent
	Lobbing	Vacations
		Mergers and acquisition

For more focus on probability and hazard, the risk map was developed to show the location of each risk based on its probability and hazard. This map was divided into 16 areas based on 4 divisions on each axis. The risks that are located in 4 extreme areas on the corner of the map have been reported in the table 10 as risks Probability-Hazard chart.

*High Probability/High Hazard*: sanction, cash flow, information flow and ordering cycle time are top priorities to manage and should be highly considered.

*High Probability/Low Hazard*: biased interpretation of regulations, government dependency and lobbing are external risks which are not really in control of pharmaceutical companies.

*Low Probability/High Hazard*: civil war, in process contamination, natural disasters, accidents and counterfeit are high impact on supply chain function if it does happen. So building infrastructures to prevent and minimizing hazards are highly noticeable issue.

*Low Probability/Low Hazard*: human resource turnover, supplier agent, vacations and mergers and acquisition are considered low priority risks for pharmaceutical supply chain management.

## Discussion

The top risks were t financial and economic parameters, politics and then the government. The result of this study shows pharmaceutical industry and supply chain were affected by political condition of Iran, in the study period. In addition, based on expert opinions, financial function is the first priority to manage in the pharmaceutical industry with a priority of 0.380 and this result is inline with the top rank risks identified.

Political status and related risks forcing companies to focus on financial and supply management so quality issues are the last priority to manage. It could contribute to compromising quality which is one of the primary objectives of supply chain management and national drug policy in Iran.

Fifty one out of 86 risk items were external and after ranking it was also observed that top 20 risks were also external. But average weight and probability of internal and external risks have no significant differences statistically. It stands for that half of the total risks in the pharmaceutical supply chain in Iran are internal risks which could be mitigated by companies, internally.

It is interesting to note that this work carried out when Iran was facing rigorous international sanctions. Since the method of risk identification and hazard assessment were based on expert judgment; hence political issues and related risks may get a higher grade than others especially in the case of normal conditions. Also challenges which each expert had faced at the time of field could affect on highlighting or dismissing some items during questionnaire responding.

Considering most important risks affecting on each function, companies could draw mitigation plan to minimize top rank risks based on company’s strategies.

The result shows, top rank risks with high effect on financial management and supply management are closely similar to top rank risks in the pharmaceutical supply chain. But ranking in operation management and quality management are different. These results confirm the result of AHP method that showed financial and supply management are extremely challenging. Operation standers, processes and human resource issues becoming in top rank risks with high effect on quality management and operation management which most of them are internal risks that could be managed inter company. And operation and quality management are affecting internal risk more than external ones and could be easier to manage.

## Conclusions

After financial and supply risks which most of them are external issues, the top risk identified in pharmaceutical supply chain include regulation issues. As one of the most significant goals of MOH is to support supply of medicine, also not addressing those risks could potentially harm supply chain. Then efficient relationship between MOH and the pharmaceutical industry could help both MOH and industry to review and mitigate regulatory risks.
